# Network Pharmacology and Molecular Docking on the Molecular Mechanism of Luo-hua-zi-zhu (LHZZ) Granule in the Prevention and Treatment of Bowel Precancerous Lesions

**DOI:** 10.3389/fphar.2021.629021

**Published:** 2021-02-15

**Authors:** Cui Guo, Xingdong Kang, Fang Cao, Jian Yang, Yimin Xu, Xiaoqiang Liu, Yuan Li, Xiumei Ma, Xiaoling Fu

**Affiliations:** ^1^ Second Department of Oncology, Yueyang Hospital of Integrated Traditional Chinese and Western Medicine, Shanghai University of Traditional Chinese Medicine, Shanghai, China; ^2^ Liaoning University of Traditional Chinese Medicine, Shenyang, China; ^3^ School of Chinese Materia Medica, Nanjing University of Chinese Medicine, Shanghai, China; ^4^ Jiangxi University of Traditional Chinese Medicine, Nanchang, China; ^5^ The Second Military Medical University, Shanghai, China; ^6^ Department of Pain, Shibei Hospital, Shanghai, China; ^7^ Infection Prevention and Control Department, Yueyang Hospital of Integrated Traditional Chinese and Western Medicine, Shanghai University of Traditional Chinese Medicine, Shanghai, China; ^8^ Department of Radiotherapy, Renji Hospital, Shanghai Jiao Tong University, Shanghai, China

**Keywords:** network pharmacology, LHZZ granule, colorectal adenoma, molecular mechanisms, AKT1

## Abstract

The Luo-hua-zi-zhu (LHZZ) granule has been widely used for the treatment of colorectal adenoma (CRA), which is a precursor of colorectal cancer (CRC). However, the active components of LUZZ and its mechanism of action against CRA have not yet been elucidated. This study was designed to investigate the effect of LHZZ on CRA and explore its pharmacological mechanisms. First, a total of 24 chemical constituents were identified in the 50% aqueous methanol extract of LHZZ granule based on the mass fragment patterns and mass spectral library using the high resolution UPLC-Q-TOF MS/MS system. Subsequently, based on a network pharmacology study, 16 bioactive compounds and 28 targets of the LHZZ associated with CRA were obtained, forming a compound-target network. Molecular docking tests showed tight docking of these compounds with predicted targeted proteins. The protein–protein interaction (PPI) network identified AKT1, CASP3, TP53 and EGFR as hub targets. The Kyoto Encyclopedia of Genes and Genomes pathway network and pathway-target-compound network revealed that the apoptosis pathway was enriched by multiple signaling pathways and multiple targets, including the hub targets. Finally, the reliability of the core targets was evaluated using molecular docking technology and *in vitro* studies. Our study indicated that the LHZZ particle has preventive and treatment effect on colorectal adenoma through multi-component, multi-target and multi-pathway.

## Introduction

Colorectal adenoma (CRA) is the precancerous lesion of colorectal cancer (CRC) (Chen et al., 2020). CRA is characterized with abnormal crypt foci in the intestinal wall, increased inflammatory exudation and proliferation of mucosal epithelial cells in local intestinal tissue (Cho et al., 2006). The incidence of CRA is more than 30% (Dejea et al., 2018). The incidence of cancerous change of a single CRA is 20–30%, and multiple CRA is 30–80% (Chen et al., 2020). Under the theory of Traditional Chinese Medicine (TCM), CRA belongs to the category of "polyps", Polyps in Traditional Chinese medicine is a general concept, and in western medicine, polys can be divided into adenomatous polyps, hyperplastic polyps, infant polyps and so on. Endoscopic excision has become the main treatment of CRA, however, removal of CRA cannot effectively reduce the recurrence (Nishihara et al., 2013). At present, folic acid, metformin, aspirin, celecoxib and other anti-proliferation and anti-inflammatory treatment are applied in clinic as anti-CRA drugs, but the effect is not satisfactory (Liu et al., 2017; Passarelli et al., 2018; Veettil et al., 2019). Therefore, it is urgent to understand the pathogenesis of CRA and find safe and effective drugs.

*Callicarpa nudiflora* Hook, belonging to the family Verbenacase, is a perennial evergreen shrub or low arbor and its dried leaves and twigs are used as traditional Chinese herbal medicine, Luo-hua-zi-zhu (LHZZ), for the treatment of inflammation and bleeding. LHZZ granule is a Chinese patent medicine, which is included in Chinese Pharmacopoeia 2015 edition. As a drug for anti-inflammation, detoxification, convergence and hemostasis, LHZZ has been widely used in clinical treatment of inflammation caused by bacterial infection, acute infectious hepatitis, respiratory tract and gastrointestinal bleeding (Yang et al., 2021). Recent pharmacological studies suggest that (Wu et al., 2020), LHZZ contains a variety of chemical components that have pharmacological effects such as anti-cancer, anti-mutation, and promotion of immune function. They have inhibitory effects on various malignant tumors, and can improve intestinal inflammation and regulate intestinal flora (Yang et al., 2021).

**FIGURE 1:**
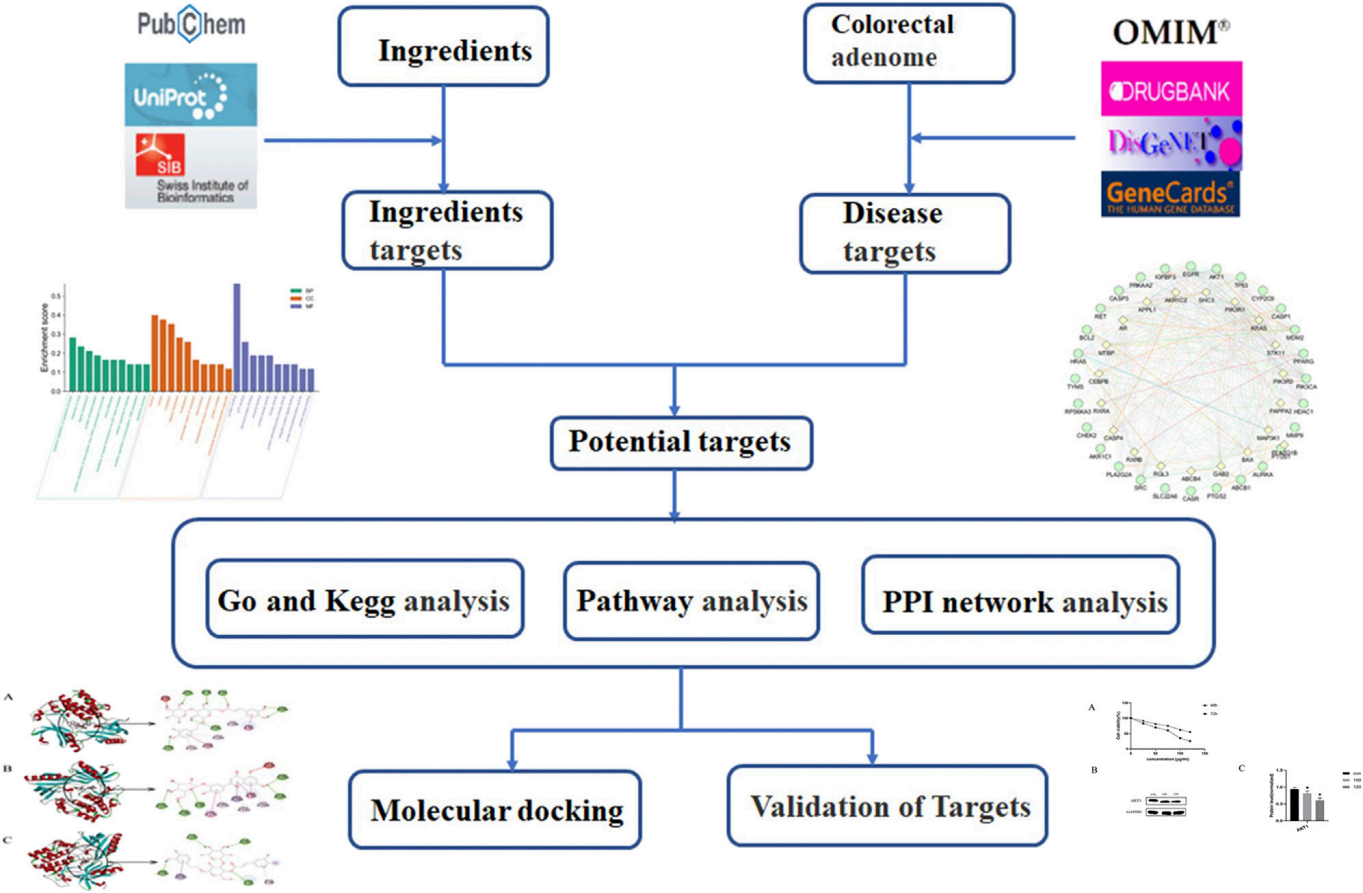
Framework based on an integration strategy of network pharmacology.

Previous studies show that LHZZ has anti-inflammatory and hemostatic functions (Wu et al., 2020), suggesting that it may be effective in the prevention and treatment of precancerous lesions of colorectal cancer. However, at present, most of the studies on LHZZ granule in the prevention and treatment of CRA are from systematic perspective, and there are few reports on the mechanism of action (Zhang et al., 2019; Yang et al., 2021). Studies based on molecular targets and related signal pathways will help to better understand the mechanism of action of LHZZ granule against precancerous lesions of colorectal cancer. Therefore, the purpose of this study is to construct a multi-dimensional network of "component-target-pathway-disease" by using network pharmacology (Boezio et al., 2017; Zhou et al., 2020) and molecular docking modality (Park et al., 2018), to explain the biological mechanism of LHZZ granule in the prevention and treatment of CRA. This study will provide a scientific basis for clinical trial research and product development of LHZZ granule. The idea of this study is shown in the flow chart of Figure 1.

## Materials and Methods

### Chemicals and Reagents

HPLC grade acetonitrile was purchased from Merck Company (Darmstadt, Germany), HPLC grade methyl tert-butyl ether from CNW, AR grade acetic acid and GR grade ethanol from Sinopharm Chemical Reagent (Shanghai, China), and Cremophor EL (BR grade) from Shanghai Yuanye (Shanghai, China). Purified water used throughout the study was prepared by a purified water system 611VP (Sartorius, Germany). LHZZ granule was provided by the Jiangxi Puzheng Pharmaceutical Co., Ltd. (Jiangxi, China; batch number: Z20060378). and stored in the laboratory of the Shanghai University of TCM.

### UPLC-MS Analysis

LHZZ granule (25 mg) was accurately weighed and placed in a round bottom flask with 25 ml 50% methanol, mixed well, sealed and soaked for 0.5 h at room temperature, and ultrasonically treated for 30 min using an ultrasonic cleaning instrument (Shenzhen, China). The extract solution was centrifuged at 14,000 rpm for 15 min at room temperature, then filtered through a 0.22-μm microporous membrane before qualitative analysis.

A Shimadzu HPLC, consisting of a CBM-20A system controller, a LC-20AD_XR_pump, a SIL-20ACXRautosampler, a CTO-10Avp column oven and a Shiseido C_18_ column (2.1 × 150mm, 2.5 µm, Shiseido Co., Ltd., Tokyo, Japan) equipped with a guard column (C_18_, 4 mm × 3.0 mm, Phenomenex Co., Ltd., Torrance, CA, United States) was used for the chromatographic separation of LHZZ granule. A linear gradient program with a mobile phase system including solvent A (0.1% Formic acid + H_2_O, v/v) and solvent B (0.1% Formic acid + acetonitrile, v/v) as follow: 95–5% A at 0.01–18 min, 5% A at 18–19 min, 5–95% A at 19–20 min, and 95% A at 20–25 min. The temperature of the analytical column and autosampler were maintained at 30°C and 4°C respectively. The total run time for a LC–MS/MS analysis was 25 min.

The instrumental settings of Q-TOF-MS/MS were as follow: ion source gas 1 (GSI) and gas 2 (GS2) were both set to 50 psi, curtain gas (CUR) was set to 40 psi, ion spray voltage floating (ISVF) was set to 3500 V in the positive mode while 3200 V was set in the negative mode, ion source temperature (TEM) was 350°C, collision energy (CE) was 60 V, collision energy spread (CES) was 15 V, declustering potential (DP) was 100 V, and nitrogen was used as a nebulizer and auxiliary gas. Samples were analyzed in both positive and negative ionization modes with a scanning mas-to-charge (m/z) range from 120 to 1,500. The quantification was performed via peak area ratio of interest analytes to IS. The Applied Biosystems Analyst version 1.5.1 software was used to control the LC–ESI/MS/MS system as well as collect and process the data.

### Target Prediction of Luo-hua-zi-zhu Active Ingredients

We obtained information on the structure of the bioactive components, including molecular structures, canonical smiles, and their ‘sdf’ files from the product databases of PubChem (https://pubchem.ncbi.nlm.nih.gov/). Then, we predicted the target of bioactive components using public databases, namely the Swiss Target Prediction and STITCH, with the species limited to ‘Homo sapiens’. Finally, we standardized the target names using the UniProtKB (https://www.uniprot.gov/).

### Construction of Compound-Target Network

The collected traditional Chinese medicine compounds and effective targets are constructed by Cytoscape 3.7.2 software (http://www.cytoscape.org/) to construct a compound-target network, The CytoNCA plugin (v2.1.6) was used to measure the topology scores of the nodes, including the betweenness, closeness, and subgraph centrality. The option “without weight” was selected (Zhang et al., 2020).

### Colorectal Adenoma Disease Target Screening

Colorectal adenomas, colon precancerous lesions and Colorectal polyp are search terms used to separately search in the OMIM database (https://www.omim.org/), the DrugBank database (http://www.drugbank.ca/), the DisGeNET database (https://www.disgenet.org/), and the GeneCards database (https://www.genecards.org/). The species “Homo sapiens” was selected and the target gene information related to CRA was collected and integrated.

### Luo-hua-zi-zhu and Colorectal Adenoma Target Screening and Network Construction

The target prediction results of the active ingredients of the LHZZ granule are matched with the search results of the CRA-related targets, and the overlapping target (Supplementary Table S1) is selected as the core target of the LHZZ granule for the treatment of CRA. The active ingredient target of the LHZZ granule and CRA target was mapped using Venny 2.1.0 (http://bioinfogp.cnb.csic.es/tools/venny/index.html) online tool. The Venn diagram was draw. A common target network was constructed using the Cytoscape.

### Construction of Protein–Protein Interaction Network (PPI) and Screening of Key Targets

The GeneMANIA tool can not only construct a PPI network, but also find a series of genes related to the input gene based on a large amount of function-related data, and analyze the interaction between them, such as co-expression and co-localization (Franz et al., 2018). In the current study, GeneMANIA is used to construct the protein-protein interaction network of the cross genes of LHZZ and CRA. Through GeneMANIA analysis, we not only obtained the relationship between the input cross genes, but also obtained the relationship with other closely related targets. In the following analysis, we call this new set of genes "predicted LHZZ targets for CRA". In order to identify the central nodes and key proteins in the PPI network, the topology parameters were calculated by NetworkAnalyzer, and the degree of centrality (betweenness, closeness, and subgraph) was determined by the CytoNCA.

### GO Function and KEGG Pathway Enrichment Analysis

The common target of LHZZ and CRA obtained by the above screening was imported into the DAVID6.8 database (https://david.ncifcrf.gov/). The species was set to be "Homo Sapiens", and GO function enrichment analysis and KEGG pathway enrichment was performed. GO functional analysis includes biological process (BP, biological process), cellular component (CC, cellular component), molecular function (MF, molecular function). It is visualized as histogram and bubble chart by the OmicShare cloud platform (http://www.omicshare.com).

### Target-Path/Functional Network Construction

Ten representative signal pathways, biological processes and molecular functions were screened for further network analysis. The target pathway/functional network was constructed by Cytoscape 3.7.2. In the network, potential targets of LHZZ for treating CRA, biological processes, and signaling pathways obtained through enrichment analysis were represented by nodes, and the interactions between them were represented by edges.

### Molecular Docking Analysis

The core compounds were screened under the condition that the "Degree" parameter of the node in the "active ingredient-target-disease" network was greater than the mean. The mol2 file of the core compound was downloaded from the TCMSP database, imported into the AutoDockTools1.5.6 software, and saved in pdbqt format. The 3D structure of the key target proteins was downloaded from PDB database (https://www.rcsb.org). The water molecules and excess inactive ligands were removed by PyMOL software. The proteins were hydrogenated and charged into AutoDockTools1.5.6 software and exported to pdbqt format. According to the published methods, the molecular docking simulation of potential targets and their corresponding components is carried out by using AutoDock vina software. The global optimal binding conformation is obtained, and the docking results are visualized by the DiscoveryStudioVisualizer.

### Experimental Verification in Vitro

#### Cell Culture

IH-CRA cells were obtained from the China Center for Type Culture Collection (CCTCC), whose storage number is C2019307. We have applied for the national invention patent, patent number: 201911261397.9 (Wuhan, China). Cells were cultured in Ham‘s F 12 nutrient medium (F12, Beyotime Biotechnology, Shanghai, China), supplemented with 10% fetal bovine serum (FBS, Zhejiang, China). Cells were cultured at 37°C and 5% CO_2_.

#### Cell Viability Assay

IH-CRA cells in the logarithmic phase were seeded at 1 × 104cells/well in 96-well plates. After incubation for 24 h, IH-CRA cells were exposed to LHZZ (0, 25, 50, 75 and 100 μg/ml). After treatment for 48 h and 72h, 20 ml of Cell Counting Kit (CCK-8) assay solution (Beyotime, Shanghai, China) was added to each well, and cells were incubated for 4 h at 37°C and 5% CO_2_. The absorbance at 450 nm was measured by a microplate reader (FLUOstar Omega, LABTECH, Offenburg, Germany). Cell survival was calculated as: absorbance/absorbance of control × 100%. The Graphpad prism 7.0 software was used to analyze and plot the data.

#### Western Blotting Assay

Total protein was obtained from treated cells in radioimmunoprecipitation assay buffer (P0013B; Beyotime, Shanghai, China) and centrifuged at 12,000 × *g* at 4°C for 15 min. Proteins were separated via sodium dodecyl-sulfate polyacrylamide gel electrophoresis and transferred onto polyvinylidene fluoride membranes (Millipore, Bedford, MA, United States). After blocking in 5% bovine serum albumin (Gen-view Scientific Inc., United States) for 1.5 h at room temperature, the membranes were incubated with primary antibodies against AKT1 (cell signal technique) at 4°C overnight. Next, the membranes were incubated with horseradish-peroxidase-conjugated secondary antibodies (proteintech) at room temperature for 1 h. The Enhanced Chemiluminescence Detection Kit (P10100; New Cell & Molecular Biotech Co., Ltd.) was used to detect and visualize protein bands.

### Statistical Analysis

All data are expressed as the mean ± standard deviation (SD) and analyzed by one-way analysis of variance (ANOVA) followed by a least-significant difference test. *p*-values of <0.05 or <0.01 were regarded as statistically significant. The statistical analysis was performed by SPSS 20.0 (SPSS Inc., NY, United States).

## Results

### Separation and Identification of Active Components of Luo-hua-zi-zhu

**FIGURE 2:**
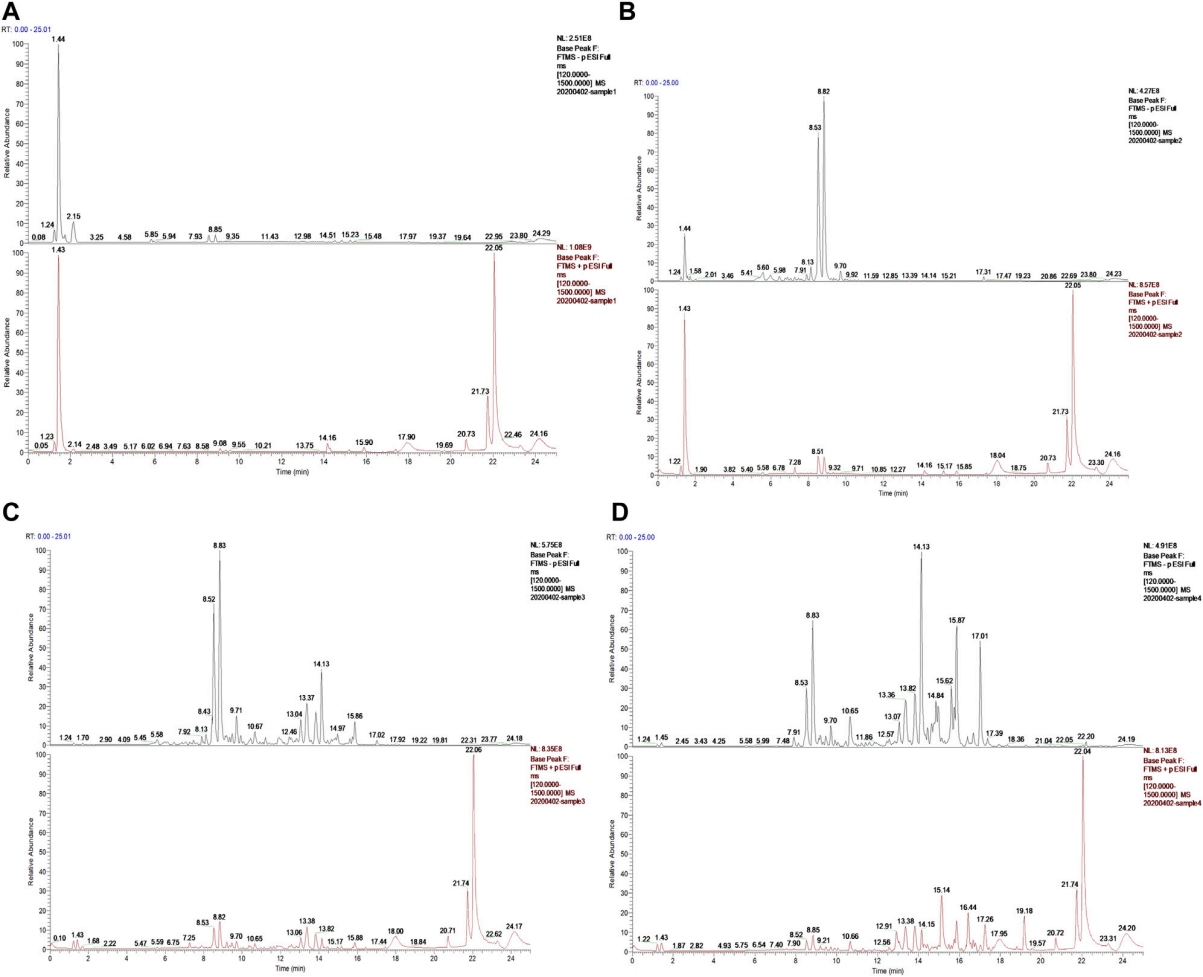
Ion chromatogram of sample separation **(A)** Water elution site, **(B)** 50% ethanol elution site, **(C)** 80% ethanol elution site, **(D)** 95% ethanol elution site.

A total of 24 chemical constituents were identified in the 50% aqueous methanol extract of LHZZ granule based on the mass fragment patterns and mass spectral library using the high resolution UPLC-Q-TOF MS/MS system. These chemical constituents include Apigenin, luteolin, and Prostaglandin B1 et al. (Table 1). The main components contain verbascoside (8.53 min) and Isoacteoside (8.82 min), of which 80% of ethanol elution has the highest content (Figure 2).

**TABLE 1:** Active ingredient of LHZZ granule.

Number	Molecular formula	PubChem CID	Molecule name	Canonical SMILES
Compound1	C20H32O5	311	(-)-Prostaglandin E2	CCCCCC(C=CC1C(CC(=O)C1CC=CCCCC(=O)O)O)O
Compound 2	C18H16O7	494583	5,4-Dihydroxy-3,7,3′-trimethoxyflavone	COC1=CC(=C2C(=C1)OC(=C(C2=O)OC)C3=CC(=C(C=C3)O)OC)O
Compound 3	C20H28O4	66789	5-[2-(Furan-3-yl)ethyl]-8a-(hydroxymethyl)-5,6-dimethyl-3,4,4a,6,7,8-hexahydronaphthalene-1-carboxylic acid	CC1CCC2(C(C1(C)CCC3=COC=C3)CCC=C2C(=O)O)CO
Compound 4	C21H20O12	185766	6-Hydroxyluteolin 7-glucoside	C1=CC(=C(C=C1C2=CC(=O)C3=C(C(=C(C=C3O2)OC4C(C(C(C(O4)CO)O)O)O)O)O)O)O
Compound 5	C17H26O11	5281788	8-O-Acetylharpagide	CC(=O)OC1(CC(C2(C1C(OC=C2)OC3C(C(C(C(O3)CO)O)O)O)O)O)C
Compound 6	C21H18O11	23928102	Apigenin 7-glucuronide	C1=CC(=CC=C1C2=CC(=O)C3=C(C=C(C=C3O2)OC4C(C(C(C(O4)C(=O)O)O)O)O)O)O
Compound 7	C15 H10 O5	31292	Apigenin	C1=CC(=CC=C1C2=CC(=O)C3=C(C=C(C=C3O2)O)O)O
Compound 8	C21H20O10	5280601	Apigenin-7-O-β-D-glucoside	C1=CC(=CC=C1C2=CC(=O)C3=C(C=C(C=C3O2)OC4C(C(C(C(O4)CO)O)O)O)O)O
Compound 9	C20H30O12	5280637	Bioside Verbasoside	CC1C(C(C(C(O1)OC2C(C(OC(C2O)OCCC3=CC(=C(C=C3)O)O)CO)O)O)O)O
Compound 10	C16H24O4	6476333	Brefeldin A	CC1CCCC=CC2CC(CC2C(C=CC(=O)O1)O)O
Compound 11	C6H8O7	5280704	Citric acid	C(C(=O)O)C(CC(=O)O)(C(=O)O)O
Compound 12	C21H20O11	5319484	Cynaroside	C1=CC(=C(C=C1C2=CC(=O)C3=C(C=C(C=C3O2)OC4C(C(C(C(O4)CO)O)O)O)O)O)O
Compound 13	C34H44O19	5280445	Forsythoside B	CC1C(C(C(C(O1)OC2C(C(OC(C2OC(=O)C=CC3=CC(=C(C=C3)O)O)COC4C(C(CO4)(CO)O)O)OCCC5=CC(=C(C=C5)O)O)O)O)O)O
Compound 14	C29H36O15	5280443	Isoacteoside	CC1C(C(C(C(O1)OC2C(C(OC(C2O)OCCC3=CC(=C(C=C3)O)O)COC(=O)C=CC4=CC(=C(C=C4)O)O)O)O)O)O
Compound 15	C21H18O12	5287620	Luteolin 7-glucuronide	C1=CC(=C(C=C1C2=CC(=O)C3=C(C=C(C=C3O2)OC4C(C(C(C(O4)C(=O)O)O)O)O)O)O)O
Compound 16	C15H10O6	5280388	Luteolin	C1=CC(=C(C=C1C2=CC(=O)C3=C(C=C(C=C3O2)O)O)O)O
Compound 17	C21H20O11	12310371	Luteolin-3”-O-β-d-glucopyranoside	C1=CC(=C(C=C1C2=CC(=O)C3=C(C=C(C=C3O2)O)O)O)OC4C(C(C(C(O4)CO)O)O)O
Compound 18	C21H20O11	5319116	Luteolin-4”-O-β-d-glucopyranoside	C1=CC(=C(C=C1C2=CC(=O)C3=C(C=C(C=C3O2)O)O)O)OC4C(C(C(C(O4)CO)O)O)O
Compound 19	C7H13NO3	12309350	N-Acetylvaline	CC(C)C(C(=O)O)NC(=O)C
Compound 20	C24H28O13	5280360	Nudifloside	C1=COC(C2C1C(C3C2(O3)CO)OC(=O)C=CC4=CC(=C(C=C4)O)O)OC5C(C(C(C(O5)CO)O)O)O
Compound 21	C29H36O16	5281677	Plantamajoside	C1=CC(=C(C=C1CCOC2C(C(C(C(O2)CO)OC(=O)C=CC3=CC(=C(C=C3)O)O)OC4C(C(C(C(O4)CO)O)O)O)O)O)O
Compound 22	C20H32O4	11754080	Prostaglandin B1	CCCCCC(C=CC1=C(C(=O)CC1)CCCCCCC(=O)O)O
Compound 23	C18H37NO	5281800	Stearamide	CCCCCCCCCCCCCCCCCC(=O)N
Compound 24	C29H36O15	12000883	Verbascoside	CC1C(C(C(C(O1)OC2C(C(OC(C2OC(=O)C=CC3=CC(=C(C=C3)O)O)CO)OCCC4=CC(=C(C=C4)O)O)O)O)O)O

The 24 components isolated from the LHZZ were analyzed by the Swiss Target Prediction database. A total of 359 potential targets were screened after removing duplicates in the target prediction. Subsequently, we used Cytoscape to construct a visual "compound-target" network with 383 nodes and 770 edges (Supplementary Table S1). Nodes represent ingredients and their corresponding targets. The higher the degree corresponding to the node, the greater the pharmacological effects of this ingredient or target (Table 2).

**TABLE 2:** Topological parameters of the compound.

Compound	Degree	Subgragh	Betweenness	Closeness
Compound7	100	225,335.1	40,899.59	0.409432
Compound22	100	46,496.66	50,719.4	0.411195
Compound10	100	46,093.81	52,926.96	0.413868
Compound2	57	98,829.13	14,967.93	0.374877
Compound5	40	25,103.93	12,349.18	0.347589
Compound9	32	24,252.75	6,896.857	0.343217
Compound8	30	63,206.41	2,961.609	0.356011
Compound16	30	44,007.79	2,837.441	0.356011
Compound20	30	11,373.15	8,202.712	0.341987
Compound19	30	1,448.521	16118.71	0.337754
Compound12	29	61,555.43	2,747.355	0.355349
Compound4	23	45,455.85	1,481.349	0.351426
Compound3	22	1,495.715	10,508.58	0.343217
Compound15	20	36,547.63	1,176.193	0.348858
Compound23	20	2,387.378	9,161.614	0.34016
Compound17	19	35864.06	875.3793	0.348222
Compound18	19	35,864.06	875.3793	0.348222
Compound14	18	11,020.76	1,517.927	0.348222
Compound6	16	23,841.64	705.9578	0.346328
Compound1	12	371.5921	747.7414	0.273443
Compound13	7	2,454.869	213.3352	0.341376
Compound21	7	2,437.616	200.8548	0.341376
Compound24	3	435.55	18.95916	0.304868

### Colorectal Adenoma Target and Intersection Target

**FIGURE 3:**
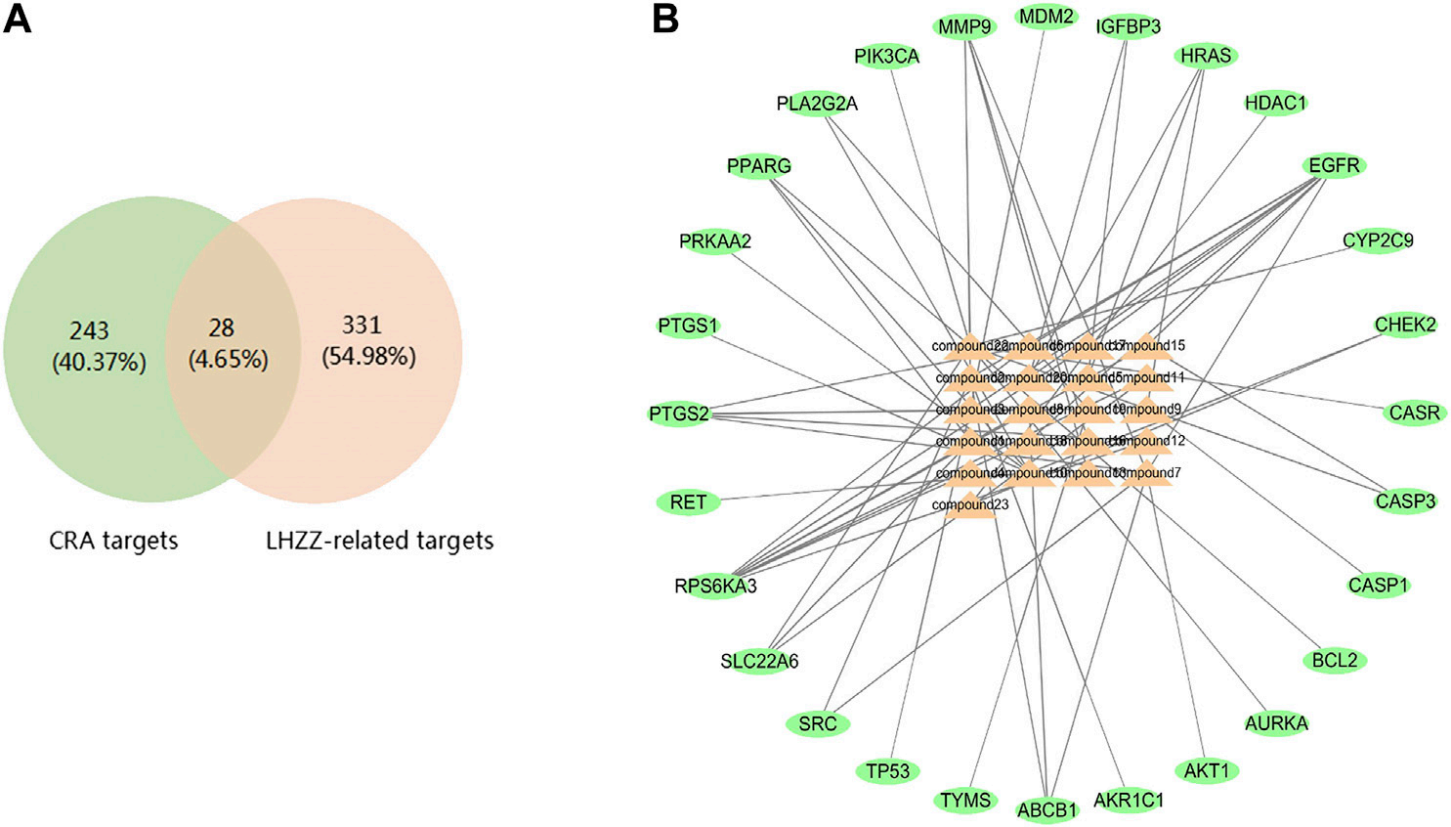
LHZZ common-target network **(A)** 28 targets are common to LHZZ and CRA, **(B)** Common-target network, including 49 nodes and 64 edges. Orange nodes stand for bioactive components from LHZZ, Green nodes stand for targets.

The intestinal adenoma-related targets were collected from the human genome database. In the OMIM, DisGeNET, DrugBank and GeneCard, with the number of these targets 173,3, 25 and 111respectively. A total of 271 LHZZ targets were obtained by removing duplicates in these four kinds of databases. These targets were intersected with component targets to obtain intersection targets 28, as shown in Figure 3A. A "component-intersection target" network diagram with 49 nodes and 64 edges was built using the Cytoscape, as shown in Figure 3B.

### Construction of Protein–Protein Interaction Network (PPI) and Key Targets

**FIGURE 4:**
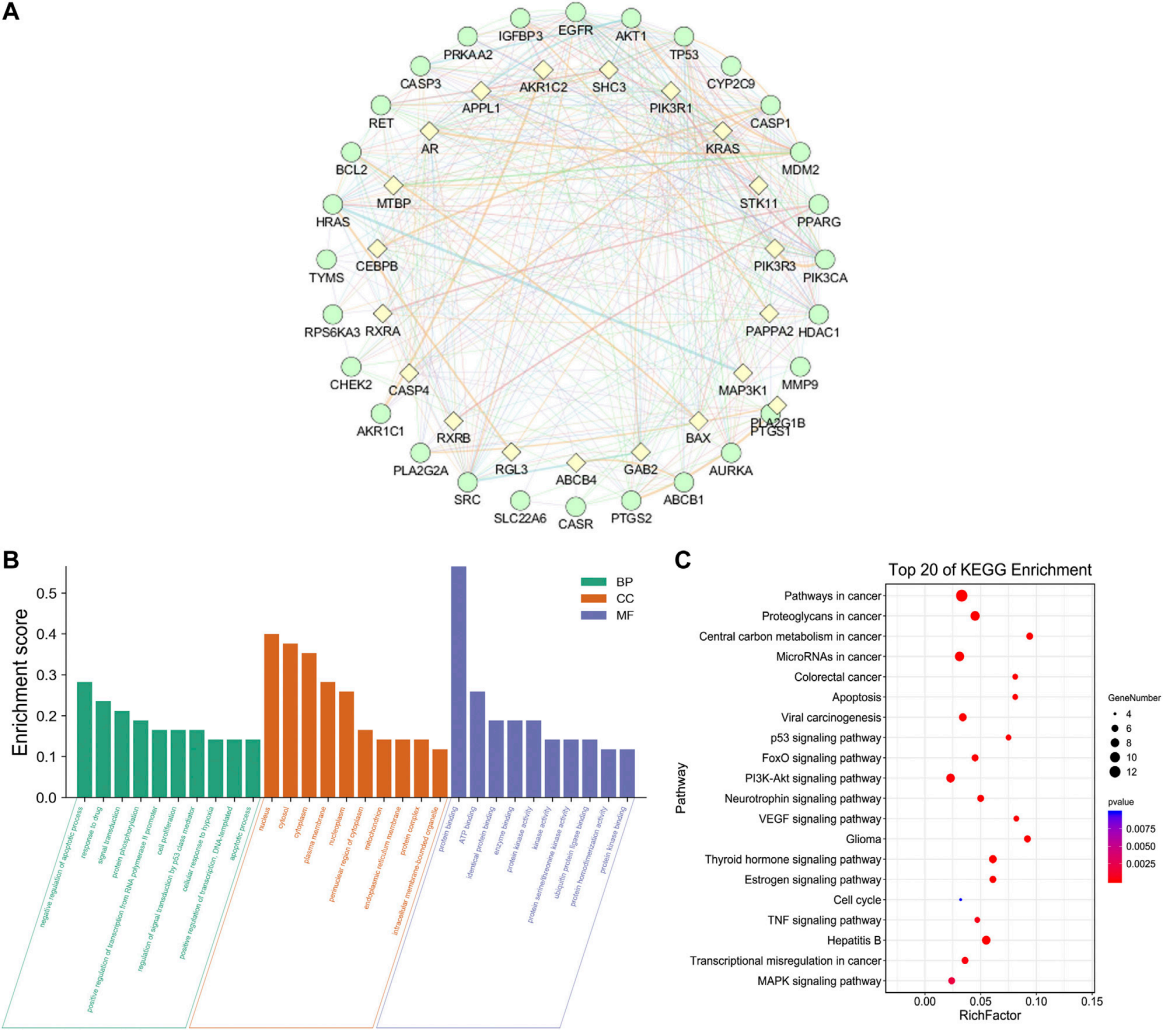
Network of potential targets of LHZZ against CRA analyzed by GeneMANIA, KEGG pathway and GO enrichment analyses of 29 target proteins (*p*-value ≤ 0.05). **(A)** Genes on the outer ring were submitted as query terms in searches. Nodes on the inner ring indicate genes associated with query genes. Functional association of targets was analyzed, and different color of connecting lines represent different correlations. **(B)** The top 10 biological processes, The top 10 molecular functions and The top 10 cellular components. **(C)** The top 20 KEGG pathways. The color scales indicate the different thresholds for the *p*-values, and the sizes of the dots represent the number of genes corresponding to each term.

In order to supplement other related genes in the network and find the key signaling pathways, we imported 28 target genes into the GeneMANIA tool to obtain a PPI network. The percentage in the results stands for the weight of interaction relationship in the network. Our result revealed that in the interaction between targets of the network, 21.82% were co-expression and 32.68% had physical interactions. There were also relationships of co-localization and shared protein domains (Figure 4A). The calculated average shortest path length, betweenness centrality, closeness centrality, and degree of nodes in the network are shown in Table 3. According to the network topology properties, the targets are sorted from high to low (degree score ≥17), corresponding to TP53, CASP3, HRAS, EGFR and AKT1. These five targets may be the key targets of LHZZ granule in the prevention and treatment of CRA.

**TABLE 3:** Topological parameters of the targets.

	Subgragh	Degree	Betweenness	Closeness
TP53	42,341.098	21	88.14873	0.83871
CASP3	40,129.74	19	39.95825	0.787879
HRAS	36,004.387	18	41.50568	0.764706
EGFR	35,467.51	17	24.41619	0.742857
AKT1	34,581.13	17	28.80135	0.742857
SRC	28,750.186	15	22.94023	0.702703
PTGS2	26,254.316	16	90.85125	0.722222
MDM2	24,656.514	13	7.926167	0.65
PPARG	23,164.822	14	34.5612	0.65
PIK3CA	18,924.707	12	54.69927	0.634146
MMP9	16,088.484	10	1.785714	0.590909
ABCB1	14,378.387	11	68.03709	0.619048
HDAC1	14,345.226	9	1.237302	0.553192
AURKA	13,083.566	9	1.194444	0.553192
TYMS	12,997.974	9	1.65873	0.565217
IGFBP3	11,296.592	8	0.222222	0.565217
CHEK2	10,979.728	9	4.732143	0.577778
RET	9,190.687	7	0.444444	0.541667
CASP1	7,237.4453	7	5.319795	0.553192
BCL2	3,940.878	5	2	0.5
RPS6KA3	1,898.8567	3	0	0.481482
PTGS1	1,169.2216	5	6.203449	0.481482
PRKAA2	1,054.8093	3	0.566667	0.490566
CYP2C9	665.86694	4	4.789682	0.472727
PLA2G2A	297.5576	3	0	0.440678
CASR	114.1672	1	0	0.393939
SLC22A6	87.212425	1	0	0.38806

### GO Function and KEGG Pathway Enrichment Analysis

**FIGURE 5:**
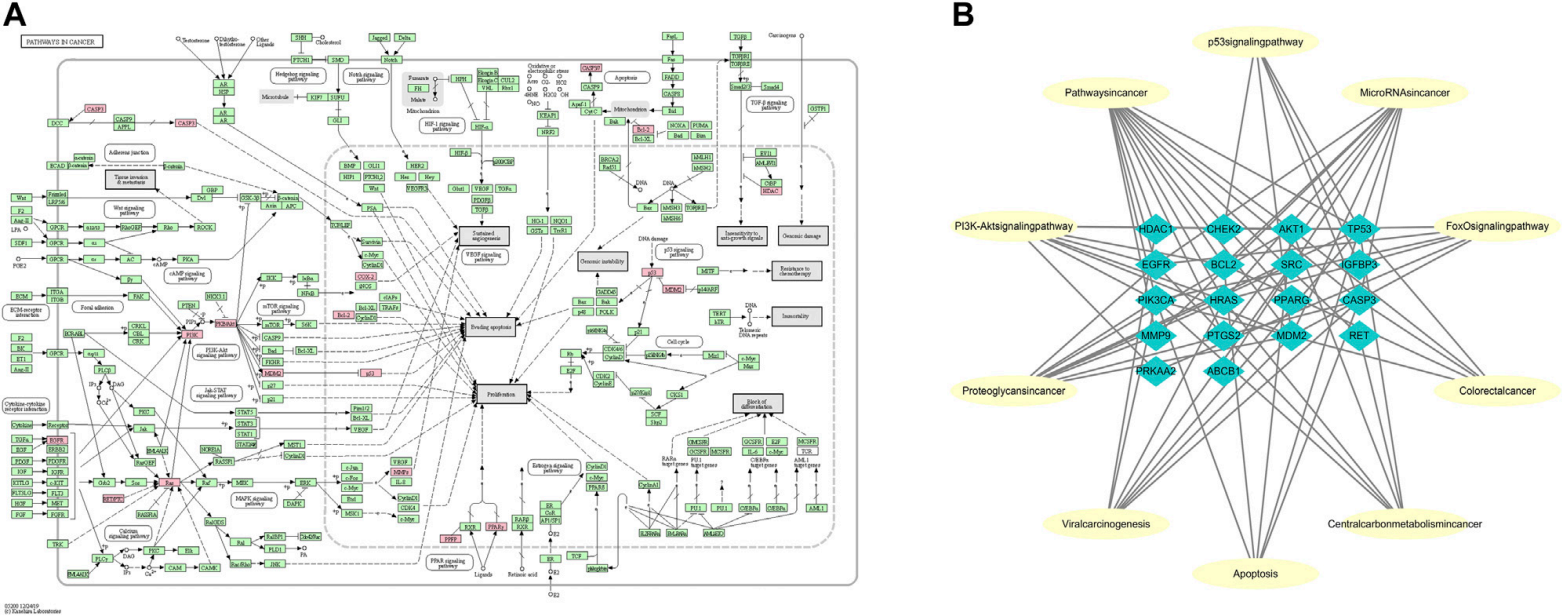
Representation of the targets of LHZZ involved in the pathway in cancer and Target-function network. **(A)** The pathway maps were constructed using the KEGG mapper. The green rectangles and red rectangles represent unidentified proteins and identified proteins, respectively. **(B)** A functional module is linked to the targets if the target is involved in that biological process or pathway.

The 28 intersected genes were enriched by GO and KEGG analysis using the analytic tool included in the David database. According to biological process (BP), molecular function (MF) and cellular component (CC) (Figure 4B), as well as *p*-value < 0.05 and gene number 6 as screening conditions, a total of 30 items related to biological process, molecular function and cellular composition were obtained. It was suggested that LHZZ may play a role in inhibiting CRA through the regulation of metabolic process, inflammatory factors, cell proliferation, protein transport, transcription factor activity and other biological processes. In the KEGG pathway enrichment analysis, a total of 71 enrichment results were obtained. Among them, cancer-related pathway, PI3K-Akt signaling pathway, p53 signaling pathway and cell apoptosis are closely associated with cell proliferation, metabolic disorder and protein transport, that are in consistent with the results of GO enrichment. KEGG pathways and gene pathways with *p* ≤ 0.05 are significantly abundant. The first 20 components are graphed using the OmicShare cloud platform **(** Figure 4C). Statistical analysis showed that five proteins participated in the first 20 pathways with a high frequency (≥9 times), which indicated that they played an important role in the enrichment pathway. The five core proteins are AKT1, TP53, CASP3, HRAS and EGFR. Among these enriched pathways, we found that cancer signaling pathways play an important role in CRA. The predictive targets of the cancer signaling pathway are shown in pink in Figure 5A.

### Target-Path/Functional Network

Based on the network analysis of several representative signaling pathways, biological processes and molecular functions, the target pathway/functional network is constructed. There are 28 nodes and 73 edges in the network graph (Figure 5B). Multiple targets participate in multiple biological processes at the same time. For example, AKT1 is involved in the processes of “cancer signaling pathway”, “PI3K-Akt signaling pathway” and “apoptosis”. TP53 belongs to both “central carbon metabolism of cancer” and “viral carcinogenesis”. EGFR participates in many biological processes, such as “MicroRNA in cancer”, “FoxO signaling pathway”, “proteoglycan in cancer” and so on. The above results suggest that LHZZ may play a role in anti-CRA through multiple targets and multiple pathways.

### Molecular Docking

**FIGURE 6:**
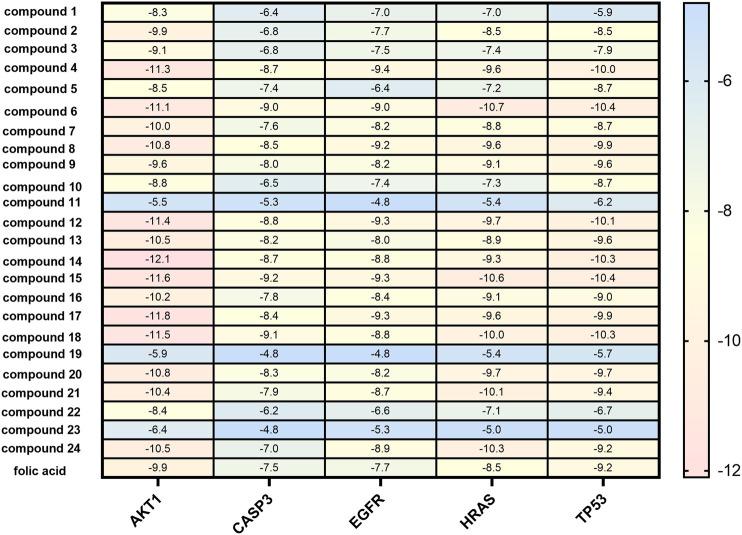
Molecular docking results heat map of key active components and key targets. The docking score changes from low to high, from blue to red. Red numbers on the right of the heat map indicate in terms of better molecular docking.

**FIGURE 7:**
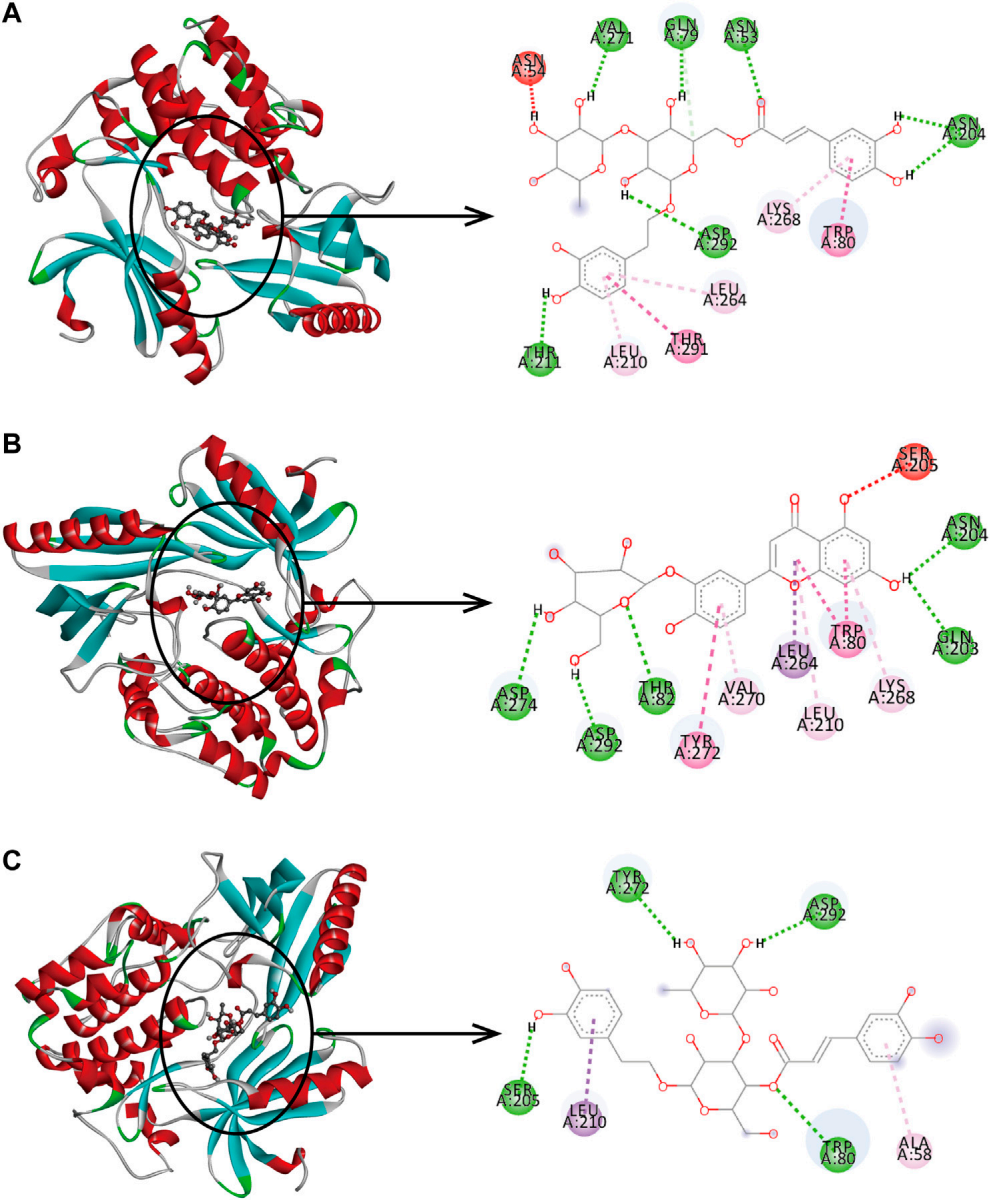
Binding pattern of core active compounds of LHZZ granule with AKT1 3D and 2D presentation of interaction between Isoacteoside. **(A)** or Luteolin-3”-O-β-d-glucopyranoside, **(B)** or verbascoside, **(C)** and AKT1.

In the network analysis of “active ingredient-target-disease”, the target of the top five proteins was docked with the active components of LHZZ granule. They were TP53, CASP3, HRAS, EGFR and AKT1. These targets were chosen because they were not only the key nodes of the PPI network, but also played important roles in KEGG signaling pathways. Folic acid was used as the positive control drug, and the score was used to evaluate the binding degree between ligand molecule and receptor molecule. The lower the score, the lower the matching energy, indicating that the more stable the conformation of ligand-receptor binding and the greater the possibility of interaction. The results showed that the common target binding energy of the active components and intestinal adenoma was negative, suggesting that the compound had a certain binding activity to the receptor, and the binding energy was less than −5 kJ/mol, indicating strong binding activity. In addition, folic acid, a positive control drug, had a therapeutic effect on CRA. The folic acid target involved in CRA therapy docked with the potential target of LHZZ, and the affinity data obtained were used as the baseline data of positive control. According to the heat map, all the 15 active components of LHZZ granule had good binding to AKT1, and their binding energies were all less than or equal to folic acid. The highest binding affinity (−12.1 kJ/mol) is Isoacteoside and AKT1, and the second highest binding affinity (−11.8 kJ/mol) is Luteolin-3”-O-β-d-glucopyranoside and AKT1 (Figure 6). Using DiscoveryStudio, the two-dimensional plan view and the three-dimensional view of the docking mode of the components and the target AKT1 are shown (Figure 7). It can be seen from Figure 7A that the binding of AKT1 to Isoacteoside is mainly through hydrogen bond interaction with VAL271, GLN79, ASN53, ASN204, ASP292 and THR211, hydrophobic interaction with LYS268, LEU264 and LEU210, and π bond interaction with TRP80 and THR291. Luteolin-3"-O-β-d-glucopyranoside binds to AKT1 (Figure 7B) mainly through hydrogen bonding with GLN203, ASP274, ASN204, ASP292 and THR82, hydrophobically with LYS268, VAI270 and LEU210, and π bond with TRP80, TYR272 and LEU264. The combination of verbascoside and AKT1 (Figure 7C) is mainly through hydrogen bonding interaction of TYR272, ASP292, SER205 and TRP80, hydrophobic interaction with ALA58 and π bond interaction with LEU210. The degree of binding between other targets and active components is interpreted in the same way as AKT1.

### Experimental Verification in Vitro

#### CCK-8 Assay

**FIGURE 8:**
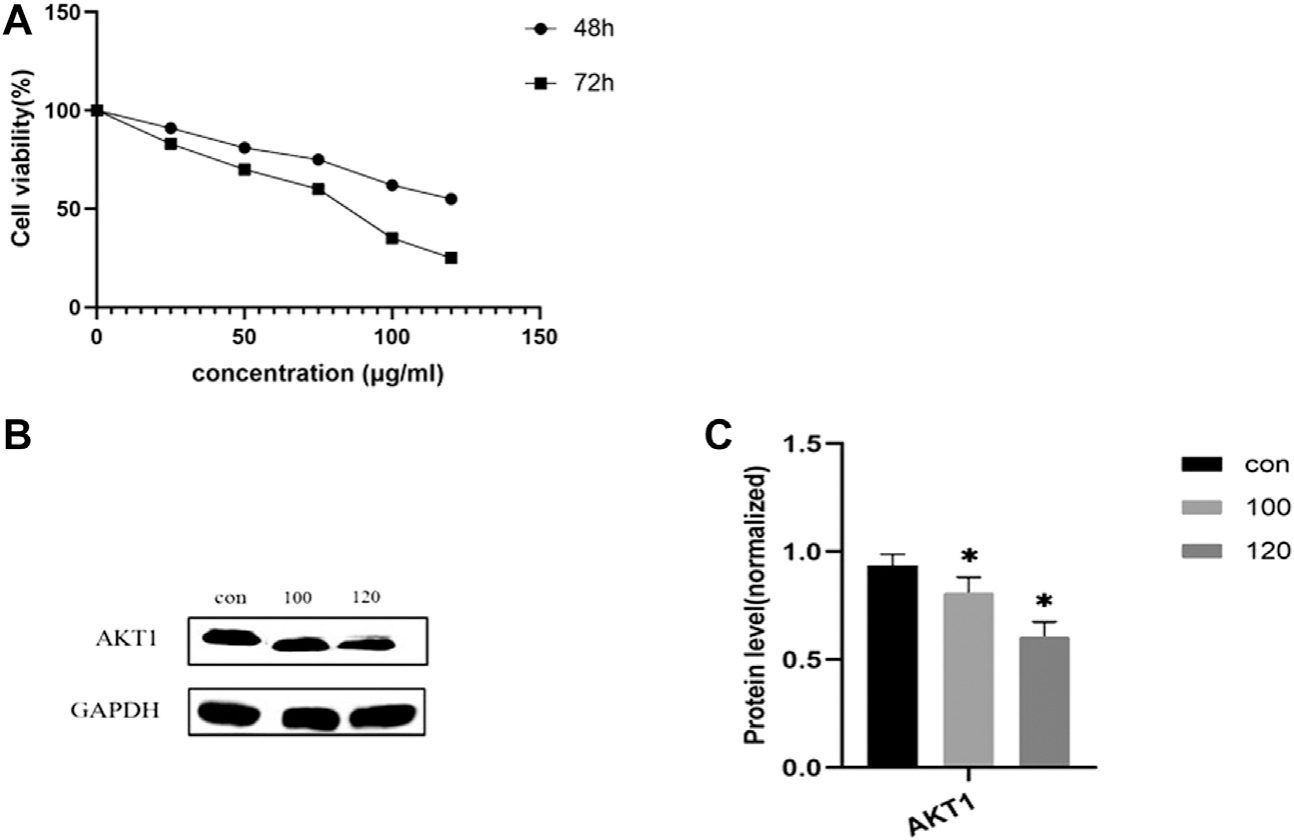
LHZZ inhibits the IH-CRA cells. **(A)** IH-CRA cells were treated with various concentrations of LHZZ for 48 and 72 h. Data are presented as the mean ± SD from at least three independent experiments. **(B)** LHZZ inhibits the IH-CRA cells. Representative Western blots showing the expression of AKT1 in IH-CRA cells. GAPDH was used as an internal control. **(C)** The protein levels of AKT1 in H-CRA cells. Values are presented as a mean ± SEM. *p < 0.05 vs. control.

CCK-8 assays were performed to determine the anti-CRA effect of treatment with LHZZ at different concentrations (0, 25, 50, 75, 100 and 120 μg/ml) for 48 and 72 h in IH-CRA cells. As shown in Figure 8A, the viability of IH-CRA cells was markedly reduced in cells treated with LHZZ in a dose- and time-dependent manner. LHZZ at ≥100 μg/ml had low cell viability (<25%).

#### Validation of Targets

We further explored the molecular mechanism of LHZZ involved in inducing apoptosis in IH-CRA cells by Western blotting. As shown in Figures 8B,C, the protein levels of AKT1 were significantly decreased in a dose-dependent manner. The results showed that LHZZ could inhibit the proliferation of IH-CRA cells and play a protective role in intestinal tissues.

## Discussions

There are approximately 1.4 million new CRC patients, and approximately 700,000 deaths worldwide each year (Cho et al., 2006). About 85% of colorectal cancer is caused by CRA. As the incidence of CRC increases year by year, the treatment and prevention of CRA has become increasingly important. CRA is the key to the pathogenesis of colorectal cancer and has the characteristics of two-way transformation, so it is the key point to prevent colorectal cancer (Sánchez-Peralta et al., 2020). According to the TCM, both the large and small intestines belong to the stomach Meridian. Polys form from the intestinal dregs and the accumulation of dampness and stasis. Therefore, the prevention and treatment of CRA contains the idea of "combined treatment on the spleen and intestine", particularly treating the spleen and stomach Meridian to treat colorectal diseases (Tang et al., 2010). The LHZZ granule can enter the spleen and stomach Meridian.

For complex diseases such as CRA, network pharmacology has unique advantages in predictive analysis (Wang and Dong, 2020). We screened the main anti-intestinal adenoma active components of LHZZ granule by network pharmacology combined with high resolution mass spectrometry analysis. We found that Apigenin, ProstaglandinB1, BrefeldinA, Isoacteoside, verbascoside and Luteolin are the main active ingredients. It was reported that apigenin and luteolin belong to flavonoids, that can inhibit the growth of intestinal adenoma cells by reducing the phosphorylation of AKT and up-regulating the expression of FADD, indicating that apigenin and luteolin can inhibit the invasion and metastasis of tumor cells (Xu et al., 2016; Kang et al., 2019). BrefeldinA is a commonly used protein transport inhibitor. Some studies have confirmed that BrefeldinA can act on the PI3K-Akt pathway and has strong anti-tumor activity (Zhou et al., 2019). As an inflammatory mediator, ProstaglandinB1 can regulate gene expression to drive the pathogenic transformation of these cells and exert its anti-inflammatory effect (Yao and Narumiya, 2019). Both Isoacteoside and verbascoside belong to phenylpropanoid glycosides and are the main components of LHZZ granule. Verbascoside is an anti-inflammatory factor, has anti-proliferation, anti-inflammatory and anti-cancer effects, that are consistent with the pharmacological action of LHZZ granule (Zhou et al., 2014; Yang et al., 2019).

The top targets in PPI network analysis are TP53, CASP3, EGFR and AKT1. TP53 is a tumor suppressor protein that can induce cell cycle arrest and apoptosis, and its mutations are common in various cancers. Some previous meta analysis show that, TP53 could be used as a biomarker for screening inflammatory bowel disease-related colorectal cancer and dysplasia (Du et al., 2017; Nakayama et al., 2017; Zhao et al., 2019). Caspase-3 is the main executor of cell apoptosis. Specific cleavage of a series of substrates such as poly-ADP ribose polymerase (PARP) and acetyl-DEVD-7-amino-4-methylcoumarin (Ac-DEVD-AMC) leads to the breakdown of DNA in cells (Peterson et al., 2009). The results of gastrointestinal tumor biomarker network analysis show that EGFR (epidermal growth factor receptor) is involved in regulating the proliferation and differentiation of trophoblast cells, and can be used as a predictive biomarker for intestinal cancers (Vaseghi Maghvan et al., 2017). AKT1 is the core member of the proliferation and survival pathway most frequently activated in cancers (Carpten et al., 2007). It was found that the upstream molecules PHLPP2 and PTEN of Akt1 affect its phosphorylation process, which in turn affects the PI3K/Akt signaling pathway, and exerts an anti-tumor effect. For further experimental verification, AKT1 was selected as a candidate target of LHZZ against CRA. The molecular docking research showed good affinity of LHZZ to these five targets, among them AKT1 has the highest binding degree. The results of molecular docking and *in vitro* validation experiments confirmed the results of network pharmacology.

According to the GO analysis of LHZZ granule on CRA, 30 biological processes were screened. These processes include those that regulate metabolic processes, inflammatory factors, protein transport, transcription factor activity, regulation of cell proliferation, et al. LHZZ may adjust the microenvironment of intestinal adenomas. In the results of the enrichment analysis of the KEGG pathway of LHZZ on CRA, the top 20 signaling pathways were screened out. Among them, the most enriched targets are cancer pathways that involve in pro-inflammatory carcinogenesis, miRNA regulatory mechanisms, PI3K-Akt signaling pathways, p53 signaling pathways, etc. According to the previous research findings (Hung et al., 2007; Tewari et al., 2019), PI3K/Akt pathway can activate PI3K by promoting Akt phosphorylation and regulate cell proliferation, thereby promoting tumor growth. The apigenin, luteolin, Isoacteoside and Brefeldin A can inhibit the progression of intestinal adenoma by affecting Akt phosphorylation, down-regulating Akt kinase and regulating PI3K-Akt pathway and inhibiting intestinal epithelial stromal transformation (Chunhua et al., 2013; Jiang et al., 2018). Dilshar et al. (Dilshara et al., 2019) have confirmed that P53-mediated oxidative stress enhances I3M-induced apoptosis of colon adenoma cells by up-regulating DR5 and inducing apoptosis in their *in vitro* experiments. P53 can prevent CRA by promoting DNA repair, cell cycle control, and programmed cell death. Studies (Granato et al., 2017) have found that verbascoside and apigenin can activate lipid metabolism pathways through the p53 pathway and participate in the apoptosis of adenoma cells. Previous studies have shown that (Mishra et al., 2016; Sabarimurugan et al., 2020), a variety of microRNAs can be used as biomarkers for the prognosis of patients with intestinal adenoma. Traditional Chinese medicine may directly or indirectly target and regulate the corresponding miRNA expression, and then restore the balance between miRNA and oncogenes or tumor suppressor genes, maintain the balance of yin and yang of the body, and achieve the effect of prevention and treatment of colorectal adenoma. Previously, berberine has been reported to reduce the risk of recurrent colorectal adenomas and polypoid lesions after polypectomy, but oral bioavailability has been poor, and LHZZ overcomes this problem (Chen et al., 2020; Wu et al., 2020). The active ingredients of LHZZ granule can treat colorectal adenoma through multiple signaling molecules and multiple signaling pathways. In view of the limitations of network pharmacology, Firstly, the medicine is traditionally prepared might have/yield different amount/concentration of the target compounds and the public databases investigated in the study are constantly updated; thus, some other bioactive ingredients and target genes may not have been included in our analysis. Further studies are warranted to examine the potential involvement of these targets.

In summary, the Apigenin, Prostaglandin, Brefeldin A, Isoacteoside, Verbascoside and luteolin are the main active components LHZZ granule. Their targets of anti-colorectal adenoma are AKT1, TP53, CASP3 and EGFR. LHZZ participates in the regulation of CRA cell proliferation through cancer pathway, metabolic pathway, PI3K-Akt pathway, p53 pathway and microRNAs. This study provides a rationale for using LHZZ for the treatment of CRA.

## Data Availability

The raw data supporting the conclusion of this article will be made available by the authors, without undue reservation.
